# Supplementary material for: Who infects whom?—Reconstructing infection chains of Mycobacterium avium ssp. paratuberculosis in an endemically infected dairy herd by use of genomic data
Source: PLoS One. 2021 May 13;16(5):e0246983. doi: 10.1371/journal.pone.0246983 (PMC8118464; doi:10.1371/journal.pone.0246983)
Supplement: S2 Fig — Boxplots with numbers of recipients produced by individual cows, by disease phenotype and scenario. (A) shedding level (0 –always faecal culture negative, 1—low, 2 –high), (B) age at first shedding (0 - ≤3 years, 1 - >3 years, 2—ante mortem negative), (C) infection progress (0—ante mortem negative, 1—non-progressor, 2—progressor), (D) serostatus (0—ELISA-negative, 1—ELISA-positive). Scenarios (from left-most to right-most column): [birth_Basic], [birth_E], [birth_S], [shed_Basic], [shed_E] and [shed_S]. (PDF) [file pone.0246983.s002.pdf]

”Who infects Whom“ - Reconstructing infection chains of *Mycobacterium avium* ssp. *paratuberculosis* in an endemically infected dairy herd by use of genomic data (Nigsch et al., 2021)

Supporting information S2 Fig.

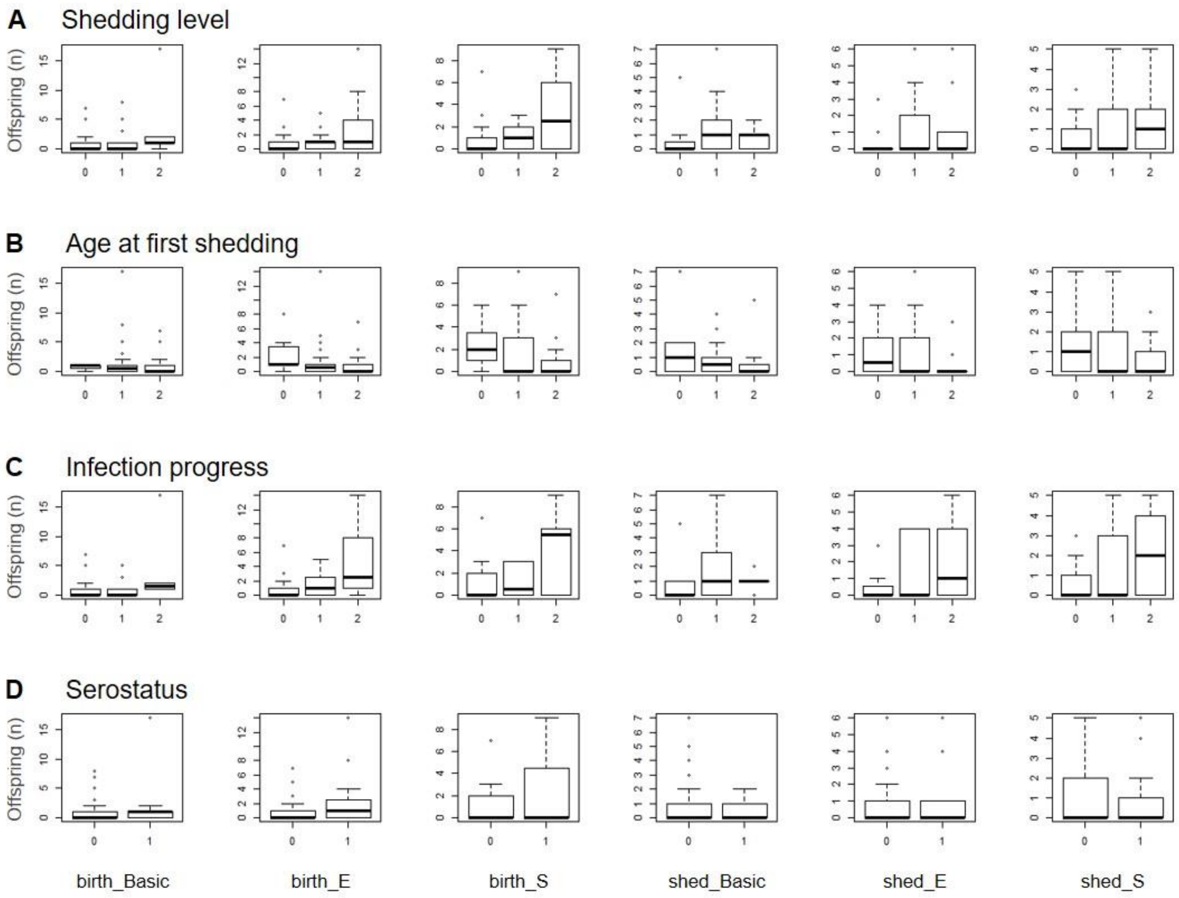

S2 Fig. Numbers of recipients produced by individual cows. Boxplots with numbers of recipients produced by individual cows, by disease phenotype and scenario. **(A)** shedding level (0 – always faecal culture negative, 1 - low, 2 – high), **(B)** age at first shedding (0 - ≤3 years, 1 - >3 years, 2 - ante mortem negative), **(C)** infection progress (0 - ante mortem negative, 1 - non-progressor, 2 - progressor), **(D)** serostatus (0 - ELISA-negative, 1 - ELISA-positive). Scenarios (from left-most to right-most column): [birth_Basic], [birth_E], [birth_S], [shed_Basic], [shed_E] and [shed_S].
